# Case Report: The first record of *Eustrongylides* sp. infection in the Chinese alligator (*Alligator sinensis*)

**DOI:** 10.3389/fvets.2025.1579738

**Published:** 2025-05-14

**Authors:** Yujun Shuai, Yongkang Zhou, Pingsi Yi, Jinhong Zhao

**Affiliations:** ^1^Department of Medical Parasitology, Wannan Medical College, Wuhu, Anhui, China; ^2^The National Nature Reserve of China Alligator in Anhui, Xuanzhou, Anhui, China; ^3^National Long-Term Scientific Research Base of Artificial Breeding and Protection of China Alligator in Anhui, Xuanzhou, Anhui, China; ^4^Anhui Province Key Laboratory of Biological Macro-molecules Research, Wannan Medical College, Wuhu, Anhui, China

**Keywords:** *Alligator sinensis*, *Eustrongylides*, nematode, molecular characteristics, China

## Abstract

Although digestive tract parasites are widely spread in wild or farmed crocodiles worldwide, only limited data are available on *Eustrongylides* sp. reported in crocodiles. The Chinese alligator (*Alligator sinensis*) is endemic to the Yangtze River in China, and only a few parasites have been reported to infect the Chinese alligator. In this study, a nematode was collected in the abdominal fascia of a captive deceased Chinese alligator. Cytochrome oxidase I (COI), internal transcribed spacer region (ITS) and partial small subunit DNA segments (18S) sequences were amplified to further confirm the genetic information of the species. The results showed that the nematode was attributed to the genus *Eustrongylides*. Overall, this is the first report of *Eustrongylides* sp. infected in the Chinese alligator, expanding the known host range of this nematode and contributing to a better understanding of its life cycle.

## 1 Introduction

The Chinese alligator, *Alligator sinensis* Fauvel (1), is the only crocodile species distributed in China, with about 200 million years of evolutionary history (1, 2). The species is classified as a first-class nationally protected wild animal in China (3) and is evaluated as a critically endangered species on the International Union for Conservation of Nature (IUCN) Red List of Threatened Species (4). The potential harm of parasites to the survival of this species is still a problem and deserves attention. In 2015, Zhao et al. (5) analyzed the diversity of parasites in the faeces of captive Chinese alligators and found five nematodes, four trematodes, and three protozoa. Next year, Zhao et al. (6) initially described a new species of nematode, *Ortleppascaris sinensis*, which infects the gastrointestinal tract in Chinese alligators. And, in 2020, Huang et al. (7) made the initial description of *Cryptosporidium* in the faeces of Chinese alligators.

The genu*s Eustrongylides* (Nematoda: Dioctophymatoidea) is generally considered to have the following three basic species: *Eustrongylides ignotus*, *Eustrongylides excisus*, and *Eustrongylides tubifex* (8). The nematode *Eustrongylides* sp. has a complex life cycle, and the first intermediate hosts are oligochaetes (phylum Annelida), the second intermediate hosts are fishes that feed on plankton, such as *Monopterus albus*, *Odontobutis obscurus*, and *Channa asiatica* (8, 9). The infective larvae (fourth-stage larvae) of *Eustrongylides* sp. are parasitic in the muscles, and visceral serosae of fish in the form of cysts, and the adults are found in the walls of the proventriculus, ventriculus, and intestine in fish-eating birds (10). The nematode *Eustrongylides* has been reported to have accidental hosts, including frogs, rabbits, snakes, crocodiles, and even humans (8, 11–13). For the crocodile hosts, Stephen et al. found the presence of *Eustrongylides* sp. in the stomach contents of *Caiman yacare* in Paraguay (14). A dietary survey of Nile crocodiles in Botswana also revealed the parasitism of *Eustrongylides* sp. (11). In Louisiana, researchers have discovered *Eustrongylides* sp. in the stomachs of American alligators, which describes a new geographic record of *Eustrongylides* sp. (15). Globally, disease, pathogenicity, and fatality due to infection with *Eustrongylides* spp. have been reported in fish (16), birds (10), and reptiles (17). Fusco et al. (18) reported an outbreak of the *Eustrongylides* spp. in Zebrafishes, and this parasite can cause high lethality rates; it was the first time to report the occurrence of *Eustrongylides* spp. parasitism in a zebrafish. In contrast, the harm of *Eustrongylides* to crocodiles is not very clear.

To our knowledge, there are no reports about *Eustrongylides* sp. parasitizing in Chinese alligators. The aim of this research was to confirm the *Eustrongylides* sp. infection in the Chinese alligator and provide a more scientific basis for understanding the life cycle of *Eustrongylides* sp.

## 2 Case description (methods and results)

### 2.1 Anatomy and morphological observations

In November 2023, a deceased female Chinese alligator, aged 8 years old, was collected from the National Nature Reserve of Chinese Alligator in Anhui, China “30°90’N, 118°77′E.” It was measured at 1.5 m in length and weighed 15.4 kg. Subsequently, a necropsy was performed to investigate the cause of death. The abdominal cavity and the gastrointestinal tract of the Chinese alligator were observed. If any parasites were found, they were sent to the laboratory of the Department of Wannan Medical College for further study.

**Figure 1:**
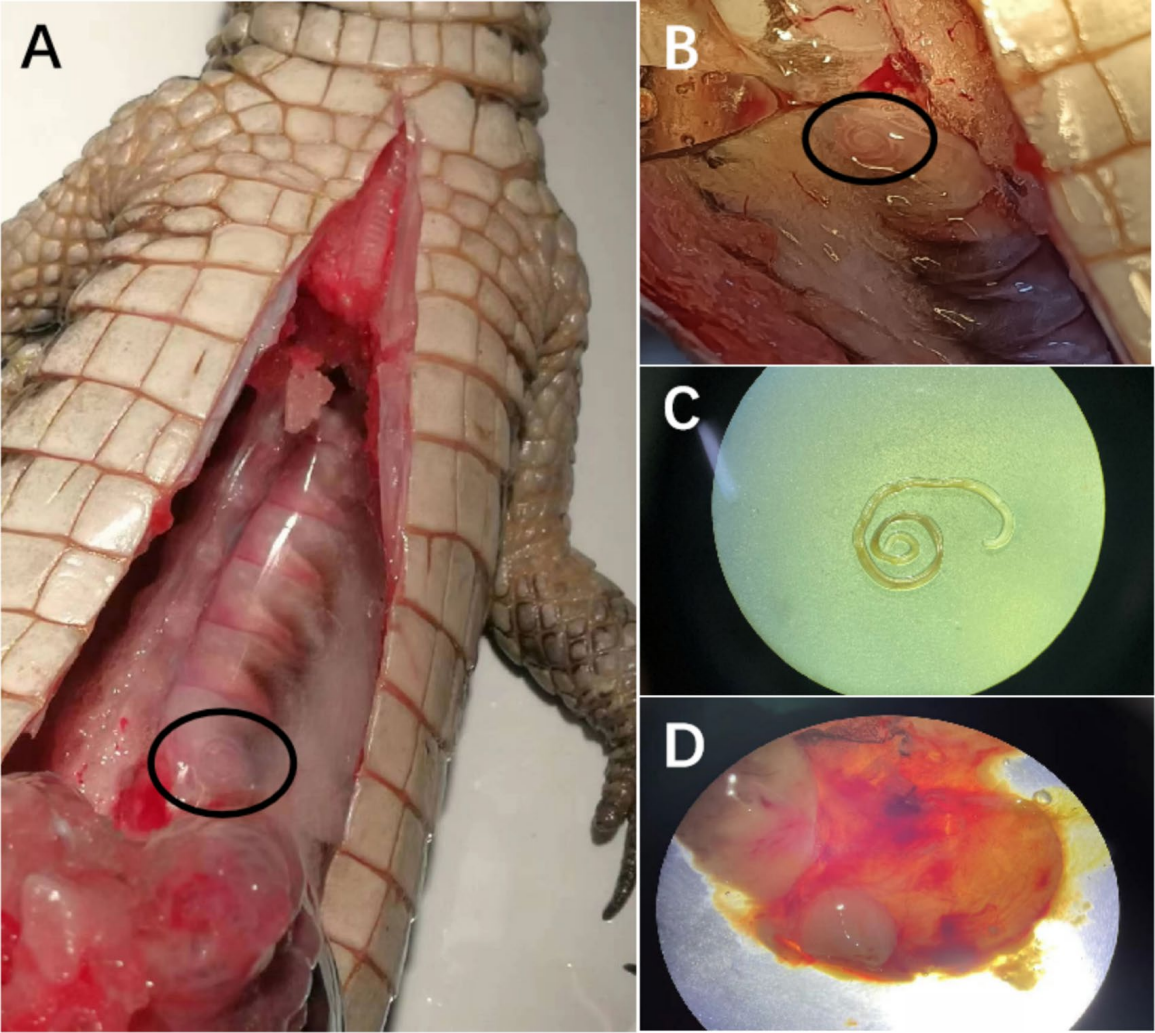
Dissection of the abdominal cavity of the dead Chinese alligator. **(A)** Spiral larva cyst in the abdominal fascia of the Chinese alligator. **(B)** Enlarged view of spiral larva cyst in the abdominal fascia of the dead Chinese alligator. **(C)** *Eustrongylides* sp. from the spiral larva cyst in the abdominal fascia of the dead Chinese alligator. **(D)** A white encysted nodule in the gastric wall of the dead Chinese alligator.

A coiled nematode was observed at the fascia of the abdominal wall from the deceased Chinese alligator (Figures 1A–C), and a white encysted nodule in the gastric wall was collected for further gastric dissection (Figure 1D). The nematode at the abdominal fascia was about 5.6 cm long, milky white, linear, and cylindrical. While we did not acquire any other nematode larvae by dissecting the white nodule under a stereomicroscope. Unfortunately, the laboratory only received a small part of the stomach and had no chance to determine whether there were other nodules in the stomach wall from the deceased Chinese alligator.

### 2.2 Molecular identification of the parasite

The nematodes were washed in physiological saline (0.9%) and then fixed in 75% ethanol. The DNA of nematode samples was extracted using a TIANamp Genomic DNA Kit (Tiangen, Beijing, China). The target genes were amplified, including the cytochrome oxidase I (COI), internal transcribed spacer region (ITS), and partial small subunit DNA segments (18S) with primers, according to Gasser et al. (19) and Floyd et al. (20) (Table 1). The primers of COI were designed in this study based on the conserved sequences in *Eustrongylides* nematodes. The Polymerase Chain Reaction (PCR) products were evaluated with a 2% agarose gel, purified with a Column DNA gel extraction kit (Sangon Biotech, Shanghai, China), and then cloned and sequenced (ABI 3730) by Sangon Biotech, Shanghai, China. The nucleotide sequences obtained were manually checked and compared using BLAST against known sequences in the GenBank DNA database. Sequence alignment and phylogenetic analysis were performed using MEGA (version X).

**Table 1:** List of PCR primers used for *Eustrongylides* sp. identification in this study.

Region	Name	Sequence (5`to 3`)	Reference
COI	COI-F COI-R	GGGCAGGAACAGGTTGAACT GAACTCAGACGAAGCAGCCT	This study
ITS	NC5 NC2	GTAGGTGAACCTGCGGAAGGATCATT TTAGTTTCTTTTCCTCCGCT	Gasser et al. (19)
18S RNA	Nem-18S-F Nem-18S-R	CGCGAATRGCTCATTACAACAGC GGGCGGTATCTGATCGCC	Floyd et al. (20)

**Figure 2:**
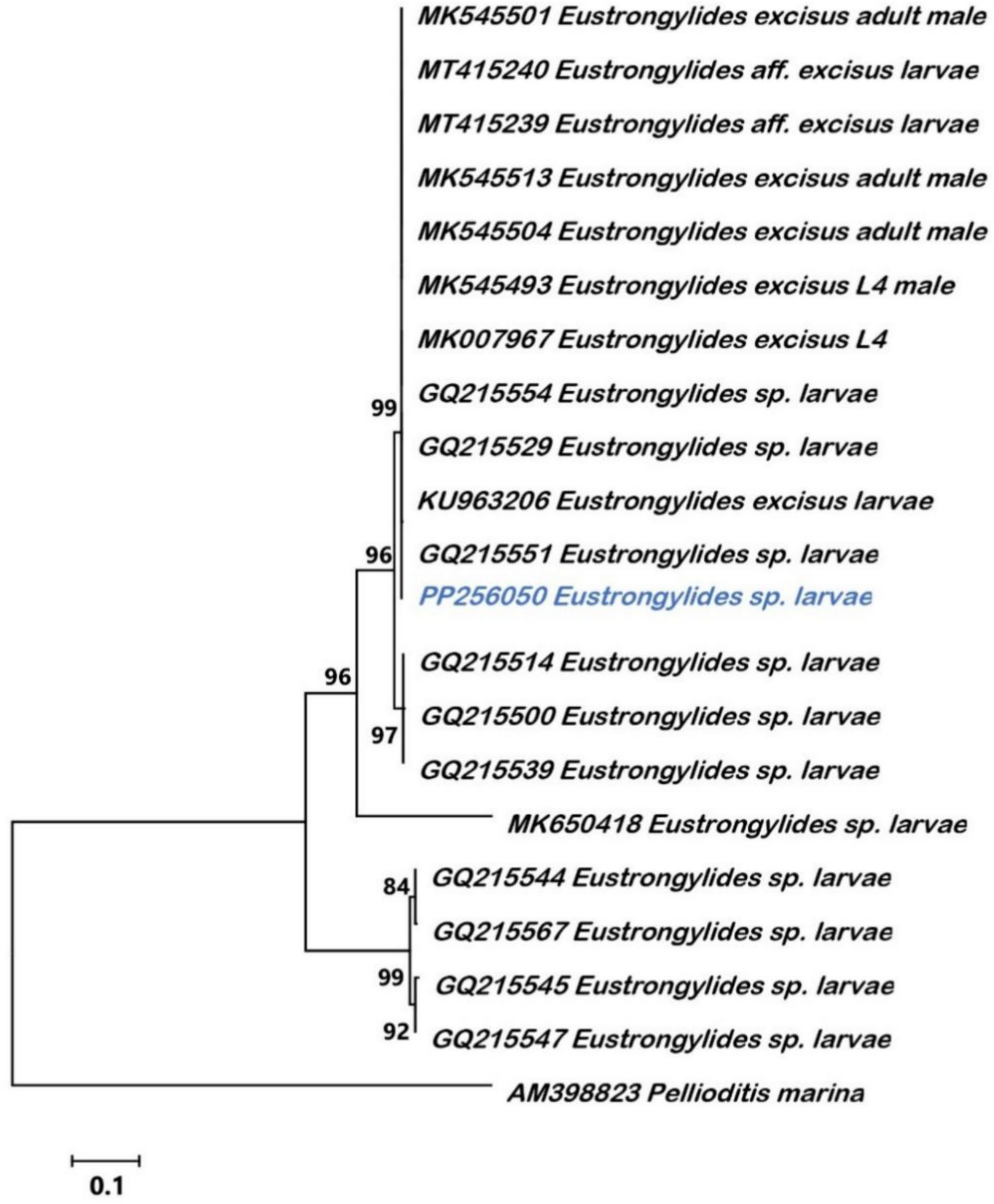
Phylogenetic relationships between *Eustrongylides* sp. by Neighbor-Joining tree obtained from ITS regions. The scale bar indicates the distance in substitutions per nucleotide. Bootstrap values were calculated over 1,000 bootstrap replicates. The blue font represents the sample of this study.

The BLAST analysis in GenBank showed that sequences of COI, ITS, and 18S rRNA genes were all attributed to the Dioctophymidae family, *Eustrongylides* genus, and the 18S rRNA had 99.12% sequence identity with the *Eustrongylides* sp. sequence (PP989425) uploaded by Iqbal. In addition, the ITS and COI sequences have 100 and 97.26% identity with the ITS (GQ215551) and COI (GQ215636) sequences of *Eustrongylides* sp. larvae identified by Xiong et al. (21) in Chinese freshwater fish, respectively. The length of COI, ITS and 18r RNA gene fragments were 467 bp, 839 bp, and 899 bp, respectively. The sequences of COI, ITS, and 18r RNA genes of *Eustrongylides* sp. were submitted to GenBank under the accession numbers PP236454, PP256050, and PP236907, respectively. Phylogenetic analysis revealed that our specimen is clustered with *Eustrongylides* sp. larvae of Xiong et al. (21) from China in a monophyletic clade with a 99% bootstrap value in ITS regions tree (Figure 2).

## 3 Discussion

Nematodes of the genus *Eustrongylides* exhibit a remarkably broad range of potential host species (8). To determine the hosts in the life cycle of the nematode *E. ignotus*, Brant et al. (22) performed the infection experiments on fishes, frogs, *Amphiuma tridactylum,* and *Alligator mississipiensis,* in which the *E. ignotus* larvae were injected orally, subcutaneously, or directly into the intestinal tract. Most predatory fishes were found hosting larvae alive in body cavities. In frogs, larvae were isolated in the abdominal wall muscles, mesentery, and lymph nodes. The *E. ignotus* larvae were subcutaneously infected into *Amphiuma tridactylum*; after a period of 42 days, one larva was located beneath the dermis, and another was identified within the muscle tissue near the injection site. In *Alligator mississipiensis*, 81 days after oral infection, necropsy revealed parasites on the abdominal wall and under the pleura (22).

In this study, we also identified *Eustrongylides* sp. in the fascia of the abdominal wall of a captive *A. sinensis*. A necropsy was performed on the dead *A. sinensis*, and the abdominal and stomach walls were observed as signs of bleeding. Cyst-like nodes (about 1.0 cm) were found in the stomach wall of the Chinese alligator. Consistent with the fundamental reaction caused by the worms in various hosts, cyst formation maintains the integrity of the parasitic structures (8, 23). Necropsies of infected birds have shown that nematodes *Eustrongylides* sp. located in the glandular stomach wall can lead to hemorrhages, ulcers and granulomas, which contain nematodes (8). Fusco et al. reported *Eustrongylides* sp. parasitism in zebrafish, causing high lethality rates because of coelomatic cavity or musculature rupture and the exteriorization of one live parasite per fish (18). While considering the crocodile hosts that have been reported, Caiman crocodile, Nile crocodile and American alligators, none of these documents mentioned that *Eustrongylides* can cause death in crocodiles. The same goes for the Chinese alligator, and it is not certain whether this *A. sinensis* died due to the nematode *Eustrongylides* sp.; the absence of additional nematodes in the coelomic cavity and stomach suggests other factors may have been involved. Furthermore, only a small portion of the stomach of the deceased *A. sinensis* was examined, which limits the scope of our findings. The cause of death likely involves a complex interplay of multiple factors, warranting further investigation. How does the Chinese alligator get infected with the parasite *Eustrongylides* sp.? In China, infection of Asian swamp eel and freshwater fish has been reported (21, 24). Xiong et al. reported the infection of *Eustrongylides* sp. in various fish species, including *Monopterus albus*, *Odontobutis obscurus*, *Channa argus*, *Elopichthys bambusa*, and *Pelteobagrus fulvidraco* within the Yangtze River, China (21). *A. sinensis* was also primarily distributed in the middle and lower reaches of the Yangtze River. In this study, the *Eustrongylides* sp. found in *A. sinensis* showed 100% identity in the ITS gene sequence with the *Eustrongylides* sp. reported by Xiong et al. (21). Chinese alligators are carnivorous reptiles that primarily hunt on fishes, frogs, birds and small mammals. Since fishes and birds serve as intermediate and definitive hosts for *Eustrongylides* sp., alligators may become accidentally infected when ingesting prey parasitized by *Eustrongylides* sp. It has not been reported that humans have been infected with this parasite, *Eustrongylides* sp., in China. However, people in some places have a habit of eating raw fish, so there may be a risk of infection.

This study enhances our understanding of the pathogenic potential and transmission mechanisms of *Eustrongylides* sp. At the same time, further research is needed to clarify the role of Chinese alligators in the life cycle of *Eustrongylides* sp. and the epidemiology of this parasite.

## Data Availability

The datasets presented in this study can be found in online repositories https://www.ncbi.nlm.nih.gov/, PP236454, PP256050 and PP236907.
